# Report of the Diseases of Edinburgh for February, 1808

**Published:** 1808-04

**Authors:** John Roberton

**Affiliations:** Surgeon


### *Report of the Diseases of Edinburgh for February,* 1808. By Mr. JOHN ROBERTON, Surgeon.

THIS month commenced with heavy falls of rain, which were almost incessant till the evening of the 3d. For twenty-four hours after this, the frost was very intense, and immediately succeeded by heavy rain, accompanied with severe gales of wind. These storms of wind, or rather hurricanes, are often experienced in this city. It has been supposed by some, that, severe as we may conceive them to be, their violence is much abated by the mountains which immediately surround Edinburgh. And here it may be necessary to mention that Edinburgh, although built on high ground, stands about the middle of a declivity commencing at the top of Pentland Hills, on the south-west, and and terminating at the shores of the Firth of Forth. Immediately on the east and south-east of the city lay Salisbury Craigs and Arthur's Seat, which is 700 feet from its base, and 814 from the level of the sea: Corstorphine Hill lays toward the west, feet high, and toward the north and north-west, in the opposite side of the Forth, the bold shores and hills of Fife appear. Having thus far sketched the geographical situation of Edinburgh, I have merely to remark concerning the previous opinion, that I not only dissent from it, but am inclined to think, that the situation of these hills causes the currents of air which are here sometimes so alarming; and for this reason in particular, that the contiguous low-laying towns of the Lothians are not infested in that way. Frost again, with some snow, commenced about the 7th, and the thermometer continued considerably below the freezing point till about the middle of the month, when the weather again became soft, and the thermometer varied from 38 to 40. Thick fogs were very prevalent from the 20th till about the 24th, when the weather became very mild and uncommonly pleasant for the season of the year; and in this way the month terminated. The barometer, except about the beginning of the month, was generally high, and toward the end of it, even during the thick fogs, it was sometimes considerably above set fair.

Catarrh still continues to prevail with considerable violence, and, perhaps, with more frequency than we have observed it in any previous winter, for at least many years. It was sometime ago believed, that this disease assumed, in many instances, the appearance of Influenza. I have, however, seen no case of this kind, nor have I been informed of its having distinctly assumed this appearance.

Very few cases of Cynanche Tonsillaris are now to be met with. I have myself seen none for the last three weeks; of course there is reason to believe, that its severity is about an end for the present.

Scarlatina Anginosa too seems of very rare occurrence. Indeed, the late appearance of this disease has been very mild.

Cases of Erysiplas are now by no means common, nor does this disease at all seem to increase either in severity or in frequency.

Inflammatory diseases in general, which have been so common and so very fatal during the whole winter, have now abated very considerably, and many of them have entirely disappeared. When the severity of these inflammatory tory diseases began to abate sometime ago, abscess in different parts of the body, particularly about the great joints, became very common, and, at present, indolent glandular swellings in different parts have occurred. I observed, in a former Report, that abscesses were probably consequences of the inflammatory diathesis which preceded their existence in the system ; and I may now remark, that it appears to me highly probable, that the glandular swellings I have just alluded to, owe their origin to the same cause.

On the present occasion, a blister applied over the affected glands has a remarkable and very speedy effect in discussing them. Before they are entirely removed, it is sometimes necessary, particularly in scrofulous habits, to repeat this application oftener than once ; but, in some cases, I have even found the blistering of the part completely inefficacious.

There is, perhaps, no disease, which not being in its ultimate consequences destructive of human life, has so puzzled both the physician and the surgeon as indolent glandular swellings. Their removal has, at all times, proved extremely tedious, and it has often been acknowledged, that the greatest ingenuity and attention were in vain exerted in attempting their removal. This is a lamentable circumstance, when we consider the great deformity which they often produce, particularly when the glands about the neck are the seat of the disease. I have seen cases where the neck, axilla, and groin were severely affected, and the only relief from the immense swellings which they occasioned, was by the slow and very partial suppuration of these enlarged glands. In some patients, whose strength was very great, this destruction of the diseased glands did not seem either very much, or very rapidly to reduce the constitution, even although it was both general and severe. When such affections, however, attacked those of weak and scrofulous habits of body, a complete cure of them was tacitly deemed impossible. The slow, gradual, but sure, exhaustion which the feelings of the decaying patient every day announced to him, as well as his general emaciation and haggard appearance, clearly demonstrated to every one what was to be the result of such a disease. Even with the strongest, the slowness of the progress of this disease completely exhausted the strength of the unfortunate patient before the destruction of all these glands had been effected. It has been asserted upon good authority, that the solutions of the muriates ates of barytes and lime, as directed in the Pharmacopeias, individually possessed great powers in discussing these glandular swellings. These medicines, however, have, I believe, fallen into disrepute, and, except by a very few individuals, entirely laid aside as apparently useless. Of the muriate of barytes, I have little, indeed nothing to say either in support or condemnation of it as an internal medicine; but, with the muriate of lime, I have been successful to a very considerable extent in removing such glandular swellings as were deemed irremovable by any other medicine with the effects of which we are at present acquainted. By repeated trials, first on myself, and afterward on others, I have found the muriate of lime, not an active dangerous medicine, as the smallness of the doses recommended by medical men would teach us to believe, but a mild and a very efficacious medicine which may be used in very large doses, and for any length of time which the obstinacy of the disease may require. In adults, after using it from a fortnight to a month, I have gradually increased the dose to an ounce per diem, and, in this ratio, continued several weeks, or even months, without any bad effects being produced, but, on the contrary, an evident diminution of the enlarged glands.

A young lady, aged 9 years, to whom I am daily administering this medicine, owing to glandular swellings about the angles of the lower jaw, and these probably of a scrofulous nature, can take half an ounce per diem without experiencing any sickness or other symptom in consequence of the doses. She first took this medicine about a year ago with the greatest advantage in the removal of her complaints. Her friends believed that she had perfectly recovered, and gave up the use of the medicine; but, for a month past, the swellings have again appeared, and she is now pursuing the same course which was formerly recommended to her. Upon the whole, I believe, that the very partial and imperfect trials that have been made with this valuable medicine, have, with the approbation of those gentlemen, who with the public, stand high in the medical profession, contributed very greatly to its being consigned to disuse. It is alone from this propensity to partiality of opinion with that unpardonable indolence which is too common in scientific pursuits, that we are to account for our present extreme ignorance respecting the nature and effects of many of our most valuable medicines. When a medicine is new, and (I may almost say) in the fashion, every person is ready to use it, and it is thus applied for the the removal of a variety of diseases for which it is not at all calculated. Disappointment, therefore, must follow such attempts, and medicines thus degraded by indiscriminate use, are first disputed by some, and, lastly, neglected by all, not because the medicines wanted powers, but because its employers wanted judgment.

It is perhaps a difficult matter to explain in what way medicine acts upon the living fibre. Many ingenious, and several ridiculous, opinions have been advanced upon this subject, and it is, without doubt, a very fair field of enquiry. A thorough knowledge of this subject, at least a greater knowledge than we at present possess, would certainly be a great acquisition to medical science, and render us capable of explaining many phenomena which at present seem to us inexplicable. There exists, at this time, a spirit of enquiry in the world, which has seldom been equalled in any former period. This I earnestly hope will lead to some advancement in the developement of this important secret. Those mere names which have in every age obstructed scientific researches, and on which all reasoning upon these subjects was founded, will I trust be laid aside, and no terms made use of in the proof of a fact but such as can be more easily explained than the fact itself. This is the only mode in which we can conduct useful reasoning, and in this way alone are we to expect to draw conclusions, whose bases, hypotheses, and useless, though often splendid, theories will never shake. With respect, however, to the muriate of lime, I may observe, that there often exists a peculiarity of action in some of these glandular swellings, rendering them extremely dense and completely irremovable, by any applications externally or internally, with which we are at present acquainted. I have seen such cases; and even the most liberal use of the muriate of lime, with the external application of blisters to the affected parts, have proved ineffectual. On the first opportunity I propose, in such cases, to administer cantharides internally, till a very slight degree of inflammation and pain is felt in the glands, when I shall discontinue the cantharides, and substitute muriate of lime for it. This action in the part, being induced by the stimulant qualities of the cantharides, the muriate of lime will then have a greater chance of exerting its influence, and consequently of removing the affection, than it could be expected to have, while the glands continued in that state of indolence which has been alluded to.

About the middle of the month, the measles, which from thei their commencement had proved, perhaps, more destructive than almost any former epidemic, began to abate in frequency, which induced us to hope that the disease had exhausted itself, and that their fatality was for the present near at an end. About a week after, however, they seemed again to gain ground upon us, and toward the end of the month, they again began to abate in frequency. I may observe, however, that, during the last three weeks of the month, when the disease fluctuated in the way above mentioned, the severity of those cases which did occur, was in general much greater than at any period during the whole winter. This was a great disappointment to us, as we expected, that, as the spring and summer approached, the disease, as on former occasions, would become milder. The two previous epidemics of this kind in Edinburgh, viz. in 1799 and in 1804, gave us reason to expect this favourable change. The first occurred in the winter, when many hundreds died ; the last during the summer months, when scarcely any died. At both these periods, the severity of the disease, as well as the treatment adopted, seemed nearly the same; consequently, the state of the atmosphere alone must have had a very great share in causing the difference in its effects.

When the Chin-cough, which was so very prevalent some time ago, began to abate in Edinburgh, it commenced in the neighbouring country, and in many instances proved fatal. Nearly the same circumstances have attended the measles at this time. While this epidemic existed here, the inhabitants of the neighbouring towns were likewise affected by it, but not severely till within these ten or twelve days, when it has similarly raged with very considerable violence.

Toward the end of the month, a sort of Typhus fever, with a petechial eruption over the body, prevailed, particularly among the lower orders of society, which, independent of every attention that could be paid to it, frequently proved fatal. No treatment, however active, seemed of much service, either in rendering it milder, or less destructive in its consequences, unless those affected with it were instantly removed from those miserable hovels in which they existed, and where they were obliged, from local situation, to breathe an atmosphere highly destructive of human life. I therefore regularly order the patients to be carried to the Royal Infirmary as soon as symptoms of the above complaint appear, and, if this be done during the first two days, a recovery is effected ed in almost every case, without even the appearance of petechiæ on any part of the body; but when the measures are delayed till the 8th or 9th day, which, from the vulgar and stubborn prejudices of these people often happens, a complete recovery I believe has seldom, if ever, been effected.

It is strange, that in every part of the kingdom, these shocking hovels should still be suffered to exist. We know to whom we ought to look for such amendment, and at whose command they can instantly be made. Were it a mere matter of convenience to the inhabitants, and only serviceable in gratifying their luxury, the fault would not be so great; but the health and lives of thousands of individuals are constantly at stake by such omissions being persevered in. The health of individuals in every nation must be of the greatest importance to its prosperity and independence, and, were I allowed to make any distinction, I would at once pronounce, that the health of the lower orders of the people is of the very greatest importance. It is from that both our army and navy, the supports of our country, receive their supplies, and consequently it is to them, that the higher orders of society owe their liberties and their privileges. It is therefore not one of the least of the duties of our government to exert some of its influence to prevent that part of the community from having their constitutions blasted by such sources of contamination, and the original vigour of their minds destroyed by diseases which, for the most part, can be so easily prevented.

The common filth and nastiness in and about the city of Edinburgh have been proverbial all over Europe, from the very earliest periods of its history; and although it has of late years been rendered much less a cause of complaint, it can by no means be said to be nearly removed. Permanent sources of nastinesss, and consequently of disease most destructive of human life, are very common, and there seems very little appearance of their being speedily removed. People who have been absent from Edinburgh a number of years allow, that the general appearance of our city is much improved, but that our filth and nastiness are not much altered for the better. This is unpardonably shameful. Why is there no tax levied against those who transgress in this way, and a person appointed to see this duty collected?

The small-pox have abated in their violence, but not by any steps that were taken to prevent their propagation.

*No. 4, St. James's Street.*

